# MSFE-GallNet-X: a multi-scale feature extraction-based CNN Model for gallbladder disease analysis with enhanced explainability

**DOI:** 10.1186/s12880-025-01902-y

**Published:** 2025-08-30

**Authors:** Hadiur Rahman Nabil, Istyak Ahmed, Aritra Das, M. F. Mridha, Md Mohsin Kabir, Zeyar Aung

**Affiliations:** 1 Department of Computer Science, American International University Bangladesh, Dhaka, Bangladesh; 2 School of Innovation, Design and Engineering, Mälardalens University, Västerås, Sweden; 3 Department of Computer Science, Khalifa University, Abu Dhabi, United Arab Emirates

**Keywords:** Image classification, CNN, Multi-scale feature extraction, Grad-CAM, Medical imaging, Ultrasound analysis

## Abstract

**Objective:**

This study introduces MSFE-GallNet-X, a domain-adaptive deep learning model utilizing multi-scale feature extraction (MSFE) to improve the classification accuracy of gallbladder diseases from grayscale ultrasound images, while integrating explainable artificial intelligence (XAI) methods to enhance clinical interpretability.

**Methods:**

We developed a convolutional neural network-based architecture that automatically learns multi-scale features from a dataset comprising 10,692 high-resolution ultrasound images from 1,782 patients, covering nine gallbladder disease classes, including gallstones, cholecystitis, and carcinoma. The model incorporated Gradient-Weighted Class Activation Mapping (Grad-CAM) and Local Interpretable Model-Agnostic Explanations (LIME) to provide visual interpretability of diagnostic predictions. Model performance was evaluated using standard metrics, including accuracy and F1 score.

**Results:**

The MSFE-GallNet-X achieved a classification accuracy of 99.63% and an F1 score of 99.50%, outperforming state-of-the-art models including VGG-19 (98.89%) and DenseNet121 (91.81%), while maintaining greater parameter efficiency, only 1·91 M parameters in gallbladder disease classification. Visualization through Grad-CAM and LIME highlighted critical image regions influencing model predictions, supporting explainability for clinical use.

**Conclusion:**

MSFE-GallNet-X demonstrates strong performance on a controlled and balanced dataset, suggesting its potential as an AI-assisted tool for clinical decision-making in gallbladder disease management.

**Clinical trial number:**

Not applicable.

## Introduction

According to the World Health Organization, gallbladder diseases represent an important public health concern, affecting millions of individuals worldwide. Gallstones, one of the most common gallbladder conditions, impact approximately 10–15% of the population in developed countries, leading to a substantial burden on healthcare systems [1, 2]. Additionally, gallbladder pathologies such as cholecystitis, adenomyomatosis, and carcinoma contribute to thousands of surgical interventions annually [3, 4]. Accurate and timely classification of gallbladder conditions is important for effective management and improved patient outcomes because misdiagnosis can lead to inappropriate treatment strategies and increased morbidity.

Traditionally, the diagnosis of gallbladder diseases has relied heavily on the manual interpretation of imaging modalities such as ultrasound and computed tomography (CT) scans. Although these imaging techniques are essential for identifying gallbladder pathologies, they can be subjective and prone to human error. This subjectivity often results in misdiagnosis or delayed treatment, particularly for complex conditions such as membranous and gangrenous cholecystitis, where the clinical presentation can be ambiguous and overlap with other abdominal issues [5]. Recent advancements in artificial intelligence (AI), particularly deep learning (DL) techniques, have shown great promise for enhancing medical image analysis, including the classification of various gallbladder conditions [6, 7]. Convolutional Neural Networks (CNNs) have appeared as powerful tools capable of automatically learning intricate features from raw imaging data [8]. This capability allows CNNs to improve diagnostic accuracy and speed compared with conventional methods, thereby addressing some of the limitations associated with traditional diagnostic approaches [9].

Despite the notable success of CNNs in medical image classification, several challenges remain in the effective classification of various gallbladder diseases. Variability in lesion appearance, including differences in size, shape, and texture, poses significant challenges for accurate diagnosis. For example, distinguishing between benign polyps, cholesterol crystals, and malignant lesions such as gallbladder carcinoma [10] can be particularly difficult and often requires expert interpretation [11]. Research has demonstrated the potential of CNNs for gallbladder classification, with models achieving accuracies exceeding 90%. For instance, a recent study reported a 91% accuracy rate when using a CNN-based approach to differentiate gallstones from other pathologies [12]. However, the complexity of gallbladder lesions necessitates the development of more refined models that can effectively manage diverse presentations and improve overall diagnostic performance.

The integration of multi-scale feature extraction methods represents a promising avenue for enhancing classification performance in gallbladder disease diagnosis. By capturing features at various scales, MSFE can provide a more comprehensive representation of gallbladder diseases, thereby addressing the challenges posed by their heterogeneous nature [13]. Additionally, the incorporation of explainable AI (XAI) techniques is vital to ensure that clinicians can understand and trust the model’s predictions. This understanding eventually facilitates better clinical decision-making and enhances the overall efficacy of the diagnostic processes [14].

The fundamental contributions of this research are as follows:

- We introduced MSFE-GallNet-X, which introduces an adaptive multi-scale feature extraction module designed to enhance the classification of various gallbladder diseases, including gallstones, cholecystitis, and carcinoma, while maintaining computational efficiency and achieving higher accuracy. The integration of MSFE enables the model to capture diverse spatial and contextual features, leading to a noteworthy improvement in classification performance.
- The MSFE-GallNet-X outperformed several state-of-the-art models in terms of accuracy and efficiency on a comprehensive dataset of gallbladder images, making it a reliable tool for clinical diagnostics.
- Our framework, MSFE-GallNet-X, incorporates XAI techniques, specifically GRAD-CAM and LIME, allowing clinicians to interpret the model’s predictions, thereby improving diagnostic confidence and supporting informed treatment planning.

The remainder of this paper is categorised as follows: Section 2 related works regarding disease identification through deep learning and image processing techniques have been mentioned and analyzed. Section 3 details the methodology employed in this study, including the dataset information, model architecture, evaluation metrics, and algorithmic approaches. Section 4 discusses the results and interpretability of our research, and Sect. 5 concludes the study. Through this work, we aim to contribute to ongoing efforts in the field of medical imaging and AI, ultimately improving the diagnosis and management of gallbladder diseases.

## Related works

Gallbladder disease classification models play a vital role in the early detection and management of gallbladder diseases. With improvements in medical imaging technologies, the analysis of ultrasound and other imaging modalities has become increasingly important for diagnosing gallbladder abnormalities. Traditional methods rely heavily on the expertise of radiologists, often leading to variability in the diagnosis and challenges in identifying subtle pathological features.

Researchers have increasingly focused on applying deep-learning techniques to classify diseases using abdominal ultrasound images. For example, Obaid et al. [15] utilized deep learning models such as VGG16, InceptionV3, ResNet152, and MobileNet for gallbladder disease classification from ultrasound images. MobileNet achieved the highest accuracy of 98.35% on a dataset of 10,692 images across nine classes. Despite this strong performance, the study highlighted limitations in the processing time with deeper models, underscoring the need for computational efficiency in practical applications. Similarly, Tan et al. employed VGG16 with transfer learning to classify idiopathic inflammatory myopathies (IIMs) from augmented ultrasound images, achieving an improved accuracy of 92.23% [16]. Although not specific to gallbladder disease, this study used grayscale ultrasound images similar to those employed in gallbladder diagnosis, making its findings relevant to our domain. In another approach, Lawley et al. introduced models such as GoogLeNet and InceptionV3, which achieved a high accuracy of 83.9% in abdominal ultrasound classification, showing strong performance in identifying kidney cross-sections with accuracies of 78.6% for the right kidney and 89.7% for the left kidney [17]. Alazwari et al. [18] employed a Bidirectional Gated Recurrent Unit (BiGRU) model, combined with the artificial Gorilla troops optimizer, for gallbladder cancer detection using the GBCU ultrasound image dataset. The dataset includes three classes: normal, benign, and malignant. The proposed model demonstrated high precision-recall values across all classes, indicating robustness, although the study’s application was limited to ultrasound images and limited categories of diseases.

Furthermore, Latha et al. introduced EfficientNet-B7 with advanced data augmentation for balanced and robust breast ultrasound image classification and achieved an impressive accuracy of 99.14%. By integrating XAI techniques like Grad-CAM, their model enhances interpretability, highlighting important features that influence classification and supporting clinical decision-making and diagnostic reliability [19]. This work, although focused on breast cancer, utilized grayscale ultrasound images, which align closely with the imaging modality used in gallbladder disease detection. Additionally, Jabeen et al. proposed a framework that integrates EfficientNet with ResNet and incorporates XAI for breast cancer classification in ultrasound images, achieving high accuracy scores of 98.4 and 98% on the BUSI dataset. Their approach includes an innovative feature selection method based on the cuckoo search algorithm, along with enhanced interpretability via XAI, which makes the model both more effective and faster than traditional deep learning methods for early cancer diagnosis [20].

Multi-scale feature extraction in image classification empowers models to capture both fine details and larger structures, which enhances their ability to handle variations in object sizes and ultimately improves accuracy. For instance, Zhang et al. developed an MM-GLCM-CNN model designed to boost lesion diagnosis by incorporating multi-scale and multi-level feature extraction from Gray-Level Co-occurrence Matrix (GLCM) descriptors. This approach strengthens the CNNs capacity to distinguish between malignant and benign lesions, even in small, pathologically-proven datasets. Their model achieved a notable AUC score of 93.%, surpassing existing methods by 1.49% [21]. Similarly, Sarkar et al. demonstrated that their MS-CNN model achieves an impressive 96.05% accuracy across seven lung disease categories from chest X-rays, excelling in multi-class classification and outperforming other advanced methods. Though these applications are outside the gallbladder domain, the use of grayscale medical images and multi-scale feature extraction strategies can directly inspire improvements in gallbladder ultrasound classification models. Basu et al. [22] proposed the FocusMAE model, a Masked Autoencoder (MAE)-based approach for detecting gallbladder cancer (GBC) from ultrasound videos. Using the Gallbladder Ultrasound Video dataset (GBUSV) with two classes (malignant and benign), FocusMAE achieved a state-of-the-art accuracy of 96.4%, surpassing previous models such as GBCNet and AdaMAE. This study noted limitations in generalizing the current methodologies to diverse data. Both studies covered a limited number of diseases. However, real-world clinical applications may require a model to distinguish between the broader spectrum of lung and gallbladder diseases. Ensemble learning approaches have gained recognition in medical imaging for their ability to enhance classification performance by combining multiple models to produce more reliable predictions. This strategy can be particularly valuable when dealing with limited labeled data, as it leverages the strengths of individual models while mitigating their weaknesses. For instance, Zhou et al. developed an ensemble method for gallbladder disease classification using sonographic grayscale ultrasound images, achieving a patient-level sensitivity of 93.1% and specificity of 93.9%, thereby outperforming human experts in diagnosing biliary atresia [23]. Similarly, Hong et al. applied an ensemble of supervised learning models to sonographic ultrasound images for gallbladder stone detection, where the random forest classifier achieved the highest accuracy of 96.33%, along with strong performance metrics such as an F1 score of 0.9636 and an AUC-ROC of 0.988 [24]. In another study, Kim et al. proposed an ensemble of three CNN models to classify true gallbladder polyps under 20 mm from ultrasound images, achieving an accuracy of 87.61% and an AUC of 0.9082 when combined with clinical features such as age and polyp size, surpassing the performance of individual models [25].

While ensemble methods offer significant benefits, they also present notable challenges. These approaches often involve increased computational cost, both during training and inference, due to the need to manage multiple models. Moreover, ensembles can become complex and difficult to interpret, making them less suitable for clinical settings where explainability is crucial. Another potential drawback is the risk of overfitting if the individual base learners are not sufficiently diverse or if the ensemble is not properly validated. Additional related studies are presented in Table 1.

**Table 1:** Some more related works

Ref.	Model	Dataset	Class	Result	Limitations
Chang et al. [26]	BPNN & GA	Shanxi Provincial People’s Hospital Dataset	3 class	sensitivity 91.72% & specificity 87.49%	$\bullet$ Limited sample size and lack of interoperability.
Loukas, C. G et al. [27]	CNN	GB image dataset	3 class	98% for 2-class classification & 83.1% for 3-class classification	$\bullet$ Single-expert assessments, dataset limitations, and class confusion affect reliability.
Reddy et al. [28]	VGG + GoogleNet + ResNet + AlexNet	Ultrasound organ images	5 class	Classification accuracy 98.77% & F-1 Scorer 98.55%	$\bullet$ Limited to single-label multi-class classification, $\bullet$ Misclassification in overlapping organ images, $\bullet$ Dependence on ultrasound image quality
Ker, J et al. [29]	DL approach	ChestX-ray 8	8 class	VoxNet 79% ResNet 80%	$\bullet$ Limited labeled datasets available. $\bullet$ Data imbalance in training sets $\bullet$ Public skepticism towards AI in healthcare.
Fujita, H et al. [30]	DL approach	Gallbladder cancer (GBC) & Xanthogranulomatous cholecystitis (XGC)	2 Class	Training accuracy 99.6% Validation accuracy 99.4%	$\bullet$ The model worked on simpler datasets. $\bullet$ Limited patient population size.
Loukas, C. et al. [31]	DL approach	GBVasc181	5 class	Patch classification accuracy: 94.48% GB wall region classification accuracy: 91.16%	$\bullet$Lack of consensus on vascularity scoring. $\bullet$ Confusion in three-class scoring.

### Research gaps

1. **Overfitting and Generalization Capability:** Some studies encountered overfitting, in which models performed well on training data but struggled with new data, indicating poor generalization. To address data shortages, certain studies have employed GANs to generate synthetic images; however, the limited diversity in real-world data restricts the variability of these synthetic images, ultimately limiting the model’s robustness and applicability across broader scenarios.
2. **High Computational Resources:** Many studies have utilized an ensemble approach that required significant computational power, such as specialized hardware. This dependence on resources increases costs and may exclude smaller research groups or organizations.
3. **Lack of explainability**: Many studies have neglected to incorporate XAI. This made it challenging to interpret and trust model predictions, especially in critical applications, such as gallbladder disease diagnosis. This limitation restricts insights into model decision-making processes, potentially hindering acceptance and accountability in high-stakes fields, such as healthcare and medication.

This study introduces a domain-adaptive architecture using a CNN model with a Multi-Scale Feature Extraction module for the detailed analysis of gallbladder images. To enhance interpretability in classifying gallbladder diseases, we applied explainable AI techniques, specifically GRAD-CAM and LIME, to visualize model decisions. Extensive experiments optimized the parameters for the CNN and both GRAD-CAM and LIME components, thereby ensuring the computational efficiency and robustness of the model. This approach was rigorously tested on a renowned ultrasound dataset, demonstrating its effectiveness in providing reliable and interpretable results for gallbladder disease classification.

## Methodology

**Fig. 1:**
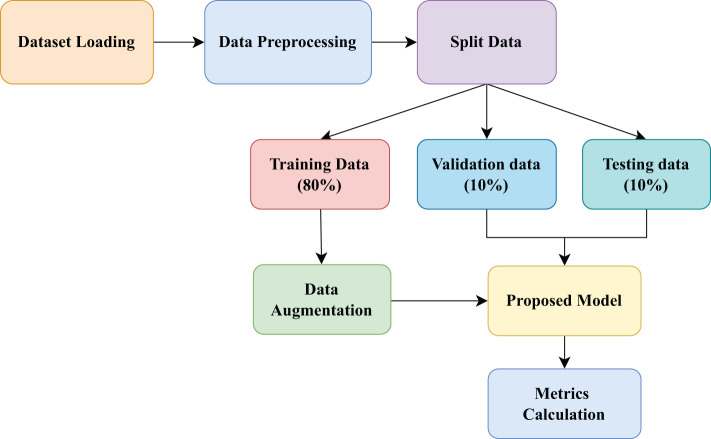
Workflow diagram of MSFE-GallNet-X

The methodology section covers the dataset acquisition, data preprocessing techniques, model architecture, hyperparameter settings, and evaluation metrics. Figure 1 shows the workflow of this study.

### Data acquisition & dataset details

**Fig. 2:**
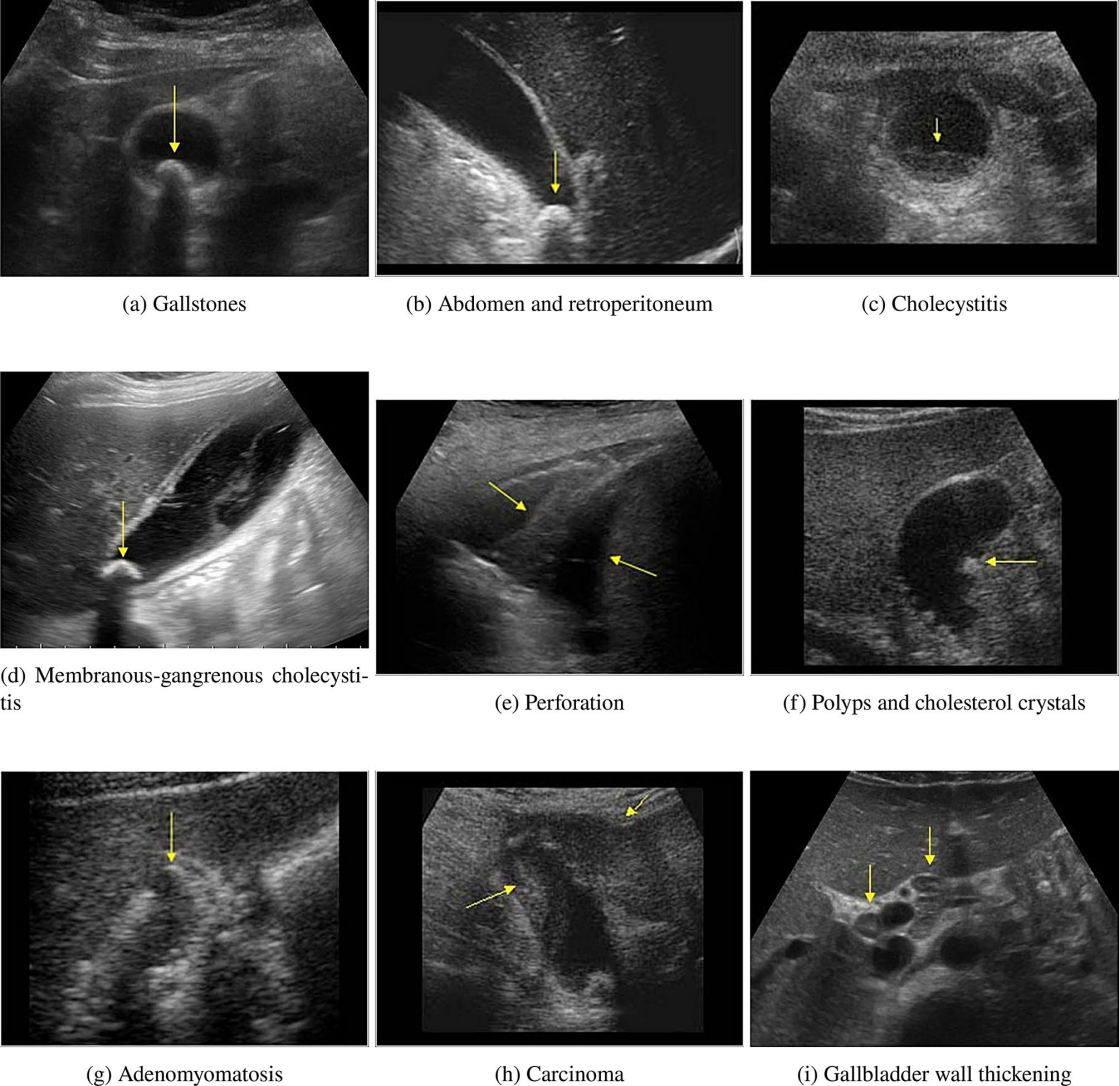
Sample images from each class in the dataset, illustrating the visual characteristics used for model training and classification

The Gallbladder Diseases Dataset offers a valuable resource for examining gallbladder diseases using ultrasound images. This dataset is significant because it addresses the void in ready-to-use medical imaging datasets that can be used to improve disease detection through machine learning and deep learning approaches. It contains 10,692 high-resolution ultrasound images collected from 1,782 individuals with a wide range of gallbladder disease spanning anatomical landmarks. Consequently, this dataset will facilitate comparative studies and the development of new analytical techniques [32]. Fig. 2 displays sample images from each class in the dataset, illustrating the visual characteristics used for the model training and classification. Table 2 shows the details of the Gallbladder diseases dataset. Table 3 presents the distribution of images across the training, testing, and validation sets (80:10:10) for each class, ensuring a balanced dataset for effective model training and evaluation.

**Table 2:** The number of images used in the dataset for training, testing, and validation

Type	No. of images
Number of images	10,692
Number of training images	8,553
Number of validation images	1,069
Number of testing images	1,069

**Table 3:** The quantity of training, testing, and validation images for each class

Class	Training	Testing	Validation
Gallstones	1061	133	132
Abdomen and retroperitoneum	936	117	117
Cholecystitis	917	115	114
Membranous and gangrenous cholecystitis	979	122	123
Perforation	850	106	106
Polyps and cholesterol crystals	816	102	102
Adenomyomatosis	931	116	117
Carcinoma	1272	159	159
Gallbladder wall thickening	792	99	99

#### Dataset quality control measures

The dataset underwent several quality control measures to ensure its reliability and suitability for gallbladder disease classification. Initially, images were classified by medical professionals at Jenin Hospital’s Specialized Gastroenterology Center, followed by a quality check by senior specialists. Ethical standards were strictly followed, with patient consent obtained and personal information removed from the images. To address inter-observer variability, a subset of images was classified by multiple experts, and the final classifications were determined through consensus. Additionally, a data quality control procedure was implemented, involving manual inspection of random image samples for anomalies and blurriness.

#### Dataset limitations

Despite the quality control measures, the dataset has certain limitations. The low contrast between the subject and the backdrop in the images, along with distortions from ultrasound scanning, can pose challenges in accurately identifying intra-abdominal organs. Furthermore, the dataset is based on 2D ultrasound imaging, which may not provide the same level of reliability as 3D ultrasound scanning.

#### Background of the classes

These nine gallbladder conditions varied in severity, prevalence, and potential complications. Gallstones, the most common condition, are hardened deposits that form within the gallbladder and can cause pain or lead to more serious issues if they obstruct the bile ducts [33]. Cholecystitis, an inflammation of the gallbladder, often results from gallstones and can be acute or chronic, causing severe pain and infection [34]. Membranous and gangrenous cholecystitis are more severe forms of cholecystitis, in which tissue death may occur, potentially leading to rupture or widespread infection [35]. Perforation occurs when the gallbladder wall ruptures, and is a medical emergency due to the risk of sepsis [36]. Polyps and cholesterol crystals are growths or deposits within the gallbladder that are generally benign but may occasionally signal early disease processes [37]. Adenomyomatosis involves thickening of the gallbladder wall and is typically benign but can mimic more serious diseases [38]. Carcinoma of the gallbladder is rare but aggressive and is the most severe condition among these, often with a poor prognosis [39]. Finally, various causes of gallbladder wall thickening may indicate inflammation, infection, or other underlying diseases, sometimes serving as early indicators of gallbladder issues [40]. Individuals affected by these conditions may experience pain, nausea, digestive issues, and, in severe cases, systemic infection or cancer, highlighting the importance of early detection and intervention in managing gallbladder health.

### Experimental setup

The experimental setup involved a comprehensive process for classifying gallbladder diseases using deep learning models. Initially, images of nine classes of gallbladder disease were collected, and the environment was set up using Google Colab with TensorFlow 2.0. Essential libraries were imported, and directories for training, testing, and validation data were structured. The proposed CNN models, both with and without multi-scale feature extraction, were developed, and transfer learning was applied using models pre-trained on the ImageNet dataset. The models were fine-tuned with a fully connected layer and softmax activation, compiled with the Adam optimizer (learning rate = 0.0001), and trained for 10 epochs. This choice of 10 epochs was based on empirical observations that performance metrics stabilized in the final few epochs, indicating convergence and making further training redundant. Early stopping was used with a patience of 3 epochs, monitoring validation accuracy. The model typically converged within 6–7 epochs. L2 regularization with a coefficient of 0.01 was applied to prevent overfitting by penalizing large weights, as it provided an effective trade-off between underfitting and overfitting in our experimental setting. A model checkpoint was configured using validation loss monitoring, and the trained model was saved. Evaluation metrics include class-wise classification reports, overall accuracy, loss curves, and confusion matrices for both the proposed and pre-trained baseline models.

### Data preprocessing

For the optimal performance of deep learning models, preprocessing enhances the quality of the input data by cleaning, transforming, and organizing it. To ensure the consistency of our dataset, the images were resized and normalized to 128 × 128pixels. This step is important for reducing noise and ensuring that the input format aligns with the model requirements. To improve generalization and prevent the model from learning any specific order in the data, the entire dataset was shuffled 10,000 times before being split into training, validation, and test sets.

#### Data augmentation

The initial pixel values were divided by 255, rescaling the intensity of each pixel in the range of (0,1). Arbitrary zooming and shearing were applied to increase the robustness of the model to slight variations in the inputs. In addition, all the images were flipped horizontally. Table 4 summarizes the data augmentation techniques used.

**Table 4:** Augmentation techniques overview

Techniques	Specifications
Rescaling	Divided by 255 and rescaled to range (0,1)
Zooming	Arbitrary
Shearing	Arbitrary
Flipping	Random Horizontal

### Proposed model

MSFE-GallNet-X is a deep learning model based on a CNN architecture that was developed for multi-class classification of gallbladder conditions. The model employed an MSFE strategy to effectively capture both fine-grained and rough patterns in medical images. Initially, we resized the images to $128 \times 128$ pixels. Subsequently, we fed the images into a 2D convolutional layer. The feature map generation through the convolutional layer follows the equation:

1 $$f[i, j] = \sum_{x}^{m_1} \sum_{y}^{m_2} \sum_{z}^{m_c} K_{[x, y, z]} \quad I_{[i + x - 1, j + y - 1, z]}$$

*where,* $m_1 = \text{height of the image}$, $m_2 = \text{width of image}$, $m_c = \text{number of channels}$, $f[i, j] =$ output value at position (*i*, *j*) after applying the filter, $K[x, y, z] =$ filter (or kernel) weight at position $(x, y, z)$, $I[i + x - 1, j + y - 1, z] =$ input value at position $(i + x - 1, j + y - 1, z)$, $x =$ index for the filter’s height, $y =$ index for the filter’s width, $z =$ channel index (depth of the filter and input).

**Fig. 3:**
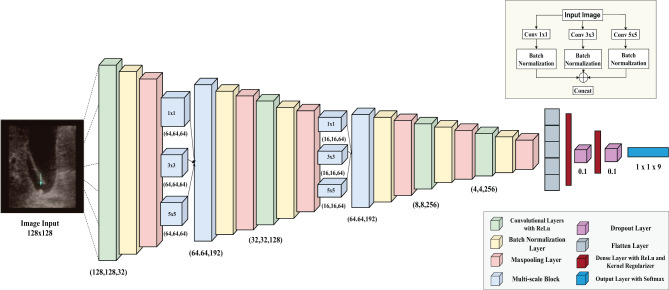
Proposed MSFE-GallNet-X model architecture

In our proposed study, MSFE uses convolutional layers with varying filter sizes of 1 × 1, 3 × 3, and 5 × 5, enabling the network to recognize features at different spatial scales. This multi-scale design is crucial for detecting subtle variations in gallbladder images. The architecture of the MSFE-GallNet-X includes six convolutional layers, with each layer followed by Batch Normalization to secure stable and efficient training. Max Pooling is applied after each convolutional layer to downsample the feature maps, thereby reducing dimensionality while preserving important information. Two of these layers incorporate multi-scale feature extraction blocks, enriching the model’s capacity to capture diverse features and enhance its overall representational power. To further improve generalization and reduce the risk of overfitting, the model incorporated L2 regularization (L2 = 0.01) within the fully connected layers and strategically applied dropout after each dense layer. The fully connected layers consist of 256 and 128 neurons, respectively, which are followed by a softmax output layer to handle multi-class classification across the nine categories of gallbladder conditions. Figure 3 illustrates the architecture of MSFE-GallNet-X, highlighting its multi-scale feature extraction blocks and the integration of various layers for effective gallbladder disease classification.

$$\operatorname{\textit{ReLU}}(x) = \begin{cases} x & \text{if}\ x > 0 \\0 & \text{if}\ x \leq 0 \end{cases}$$

In our MSFE-GallNet-X model, we integrated ReLU activation into each convolutional layer to enhance non-linearity and support efficient feature learning. In the given equation, the output returns ×when x > 0; otherwise, it returns 0. This function is commonly applied to the hidden layers of neural networks.

$$\text{Softmax}(z_i) = \frac{\displaystyle e^{z_i}}{\displaystyle \sum_{j=1}^{n} e^{z_j}}$$

Here, *z*_*i*_ is the *i*-th element of the input vector *z*, $e^{z_i}$ is the exponential of *z*_*i*_ and the denominator $\sum_{j=1}^{n} e^{z_j}$ is the sum of exponentials for all elements in *z*, which normalizes the values such that the sum of all softmax outputs is 1.

In addition, MSFE-GallNet-X is designed with Explainable AI, named Grad-CAM and LIME principles in mind, aiming to provide interpretable outputs that aid medical experts in understanding and verifying the model’s decision-making process. This combination of multi-scale feature extraction, advanced regularization techniques, and explainability ensures that MSFE-GallNet-X is both accurate and reliable in medical image analysis, making it a robust solution for distinguishing between complex gallbladder conditions.

#### Multi-scale feature extraction (MSFE) block

Multi-scale feature extraction is a crucial technique in deep learning, designed to capture details at varying spatial scales, enhancing a model’s capability to recognize both fine-grained and broad features in complex datasets. The multi-scale feature extraction block used in MSFE-GallNet-X is integrated within a domain-specific pipeline for grayscale gallbladder ultrasound imaging, with a design optimized for fine-grained lesion classification.

In our proposed model, MSFE-GallNet-X, we leveraged this approach to improve the classification accuracy for gallbladder conditions. Specifically, we integrated convolutional layers with varying kernel sizes (1 × 1, 3 × 3, and 5 × 5 filters) to extract features at multiple scales. This configuration allows MSFE-GallNet-X to effectively learn complicated patterns and slight differences in medical images by capturing detailed structures through smaller kernels while preserving larger contextual information using larger kernels. Each convolutional path is followed by batch normalization to stabilize training and enhance feature consistency. The outputs from all three branches are then concatenated along the channel dimension, forming a unified multi-scale feature representation. This design excludes pooling operations commonly used in Inception modules, to retain high-resolution spatial cues essential for ultrasound interpretation. After concatenation, an additional batch normalization and max pooling layer is applied to the merged feature map to reduce dimensionality and promote generalization. Unlike the original Inception architecture, which includes pooling and auxiliary branches optimized for RGB natural images, our MSFE block is tailored for grayscale medical images, focusing solely on spatial kernel diversity and avoiding operations that might suppress subtle diagnostic features.

**Fig. 4:**
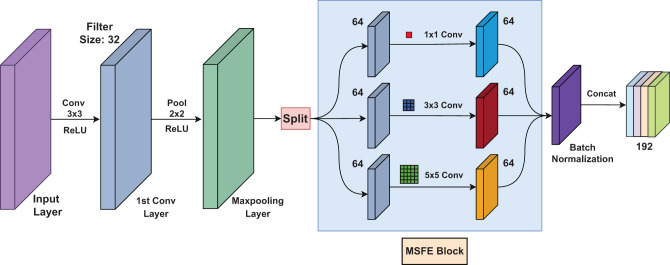
Architecture with one convolutional layer followed by a multi-scale feature extraction block

By combining these features through batch normalization and concatenation, MSFE-GallNet-X is better equipped to differentiate significant variations in gallbladder conditions, resulting in a more accurate and reliable classification framework tailored to medical imaging requirements. Figure 4 shows the multi-scale feature extraction block in the MSFE-GallNet-X.

### Hyperparameters

The hyperparameters selected for the MSFE-GallNet-X encompass the Optimizer, Dropout Rate, Learning Rate, and Decay Rate. Additional parameters include the loss function, activation functions, Image Size, Batch Size, and L2 Regularization. These settings were optimized for effective gallbladder disease classification. Table 5 presents a detailed breakdown of these hyperparameters.

**Table 5:** Hyperparameter setting for proposed model to classify gallbladder diseases

Parameter	Specification
Optimizer	Adam
Dropout Rate	0.1
Learning Rate	0.0001
Decay rate	0.2
Loss	Sparse Categorical Cross Entropy
Activation	Softmax (classification layer)
Activation	ReLU (hidden layers)
Image size	128x128
Batch size	32
L2 Regularization	0.01

### Algorithm

Algorithm 1 is developed for classifying gallbladder disease diagnosis. A publicly available Gallbladder dataset is used to test this algorithm. A deep Convolutional Neural Network model with multi-scale feature extraction layers is operated to capture both fine-grained and high-level features, thereby improving classification accuracy across various imaging conditions.

## Results and discussion

This section delivers a detailed discussion of the evaluation metrics, results, state-of-the-art comparisons, model interpretability, and overall analysis.

### Evaluation metrics

To evaluate the performance of the MSFE-GallNet-X in classifying gallbladder diseases, we used several evaluation metrics: precision, recall, F1-score, and accuracy. These metrics provide detailed insight into the model’s classification effectiveness, capturing both overall performance and class-wise performance. Additionally, the macro average and weighted average scores offer a broader perspective of the model’s performance across all classes. In the following, we describe each metric and present the calculated values.


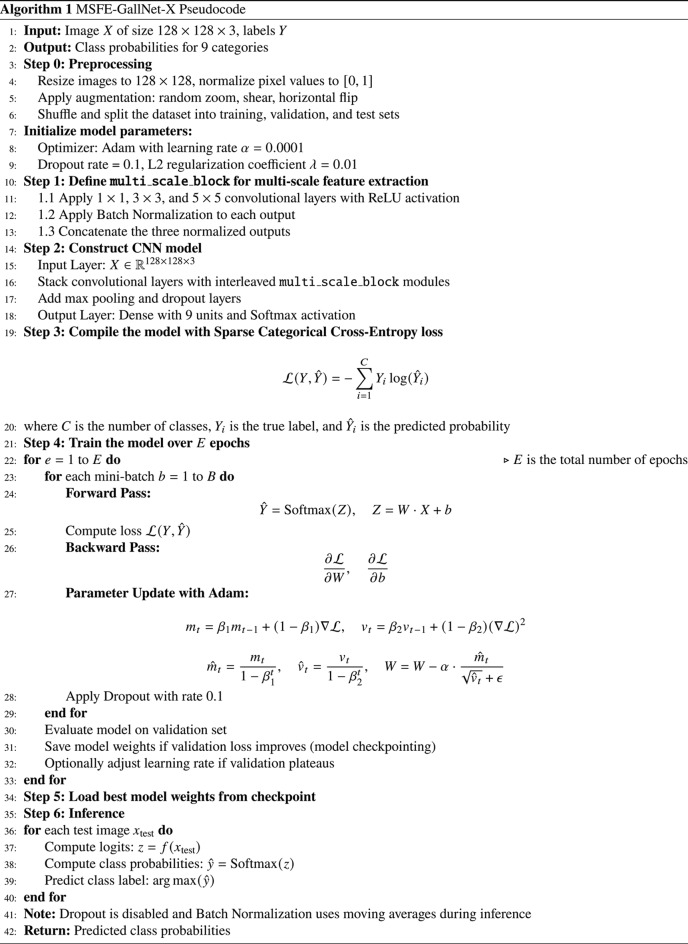


**Accuracy:** Accuracy represents the ratio of accurately predicted instances to the total instances. This provides a general measure of the effectiveness of the model across all the classes.

2 $$ \textit{Accuracy} = \frac{\textit{True Positives (TP)} + \textit{True Negatives (TN)}}{\textit{Total Number of Samples (TP + TN + FP + FN)}} $$

**Precision:** Precision indicates the accuracy of the model in identifying positive instances for each class. This is the ratio of accurately predicted positive observations to the total number of predicted positive observations.

3 $$\textit{Precision} = \frac{\textit{True Positives (TP)}}{\textit{True Positives (TP)} + \textit{False Positives (FP)}}$$

**Recall:** Recall (or sensitivity) measures a model’s ability to identify all relevant instances of a class. This is the ratio of correctly predicted positive observations to all actual observations in that class.

4 $$\textit{Recall} = \frac{\textit{True Positives (TP)}}{\textit{True Positives (TP)} + \textit{False Negatives (FN)}}$$

**F-1 Score:** The F1-score is the harmonic mean of the precision and recall, providing a single metric that balances the two. This is particularly useful when the dataset is unbalanced.

5 $$\textit{F1-Score} = 2 \times \frac{\textit{Precision} \times \textit{Recall}}{\textit{Precision} + \textit{Recall}}$$

### Results

This section discusses the accuracy, loss, precision, recall, F1 score, and confusion matrix of the proposed MSFE-GallNet-X model, along with comparisons to other prominent models.

#### Accuracy

**Fig. 5:**
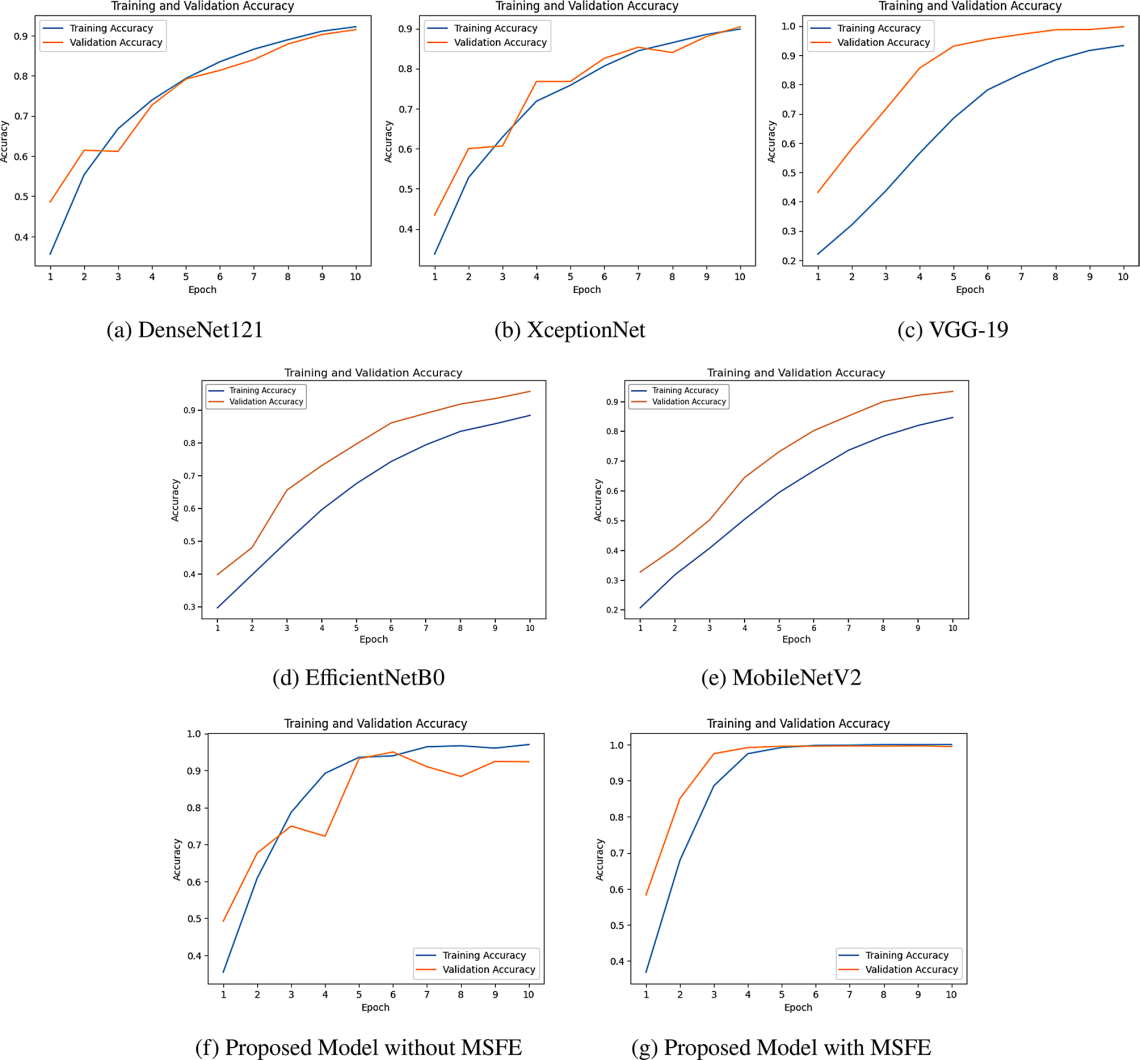
Accuracy of the models with respect to the number of epochs

To evaluate the efficiency of MSFE-GallNet-X, we conducted a comparison across different models: DenseNet121, XceptionNet, VGG-19, EfficientNetB0, MobileNetV2, GallNet-X without multi-scale feature extraction (MSFE), MSFE-GallNet-X without (w/o) data augmentation, and MSFE-GallNet-X. Each model was trained for 10 epochs, demonstrating unique accuracy trends during both training and validation phases. DenseNet121 achieved approximately 92.78% training accuracy and 91.76% validation accuracy, indicating solid performance but some limitations in generalization. However, XceptionNet performed exceptionally well, reaching nearly 90.51% training accuracy and 89.87% validation accuracy, thus demonstrating a strong fit for the task. The VGG-19 model achieved training and validation accuracies of approximately 94.04% and 99.22%, respectively. EfficientNetB0, despite being a lightweight architecture, underperformed with an overall accuracy of 75.33%, suggesting it may not effectively capture the complexity of gallbladder disease features. Conversely, MobileNetV2 achieved 93.00% test accuracy, surpassing many conventional deep models while maintaining low computational demand. Our custom MSFE-GallNet-X models yielded significantly more promising results than the pre-trained models. The base GallNet-X model without MSFE achieved a validation accuracy of 96.78%, thereby highlighting the effectiveness of our CNN-based architecture. The MSFE-GallNet-X showed even greater performance, achieving a training accuracy of 99.99% and a validation accuracy of 99.72%, which is significantly higher than the validation accuracy of GallNet-X without MSFE. This improvement underscores the impact of multi-scale feature extraction, as it enables the model to capture fine-grained features specific to this classification task. However, removing data augmentation from the proposed model reduced test accuracy by 2.77% and F1-score by 2.62%, confirming its critical role in improving generalization. The Proposed GallNet-X model without augmentation still achieved an impressive 97.0% test accuracy and F1-score, further reinforcing the model’s robustness, even in reduced training conditions. Figure 5 shows the training accuracies of the deep learning models with respect to the number of epochs.

Table 6 presents a comparison of the model performance based on testing accuracy for various architectures in gallbladder disease classification. The VGG-19 model achieved the highest accuracy of 98.89%, whereas the Proposed MSFE-GallNet-X model, which utilizes multi-scale feature extraction (MSFE), outperformed all others with an accuracy of 99.63%. DenseNet121 and XceptionNet had accuracies of 91.81% and 90.44%, respectively. Notably, the Proposed GallNet-X without MSFE demonstrated strong performance with an accuracy of 96.68%. EfficientNetB0 achieved a test accuracy of 75.33%, whereas MobileNetV2 reached 93.00%, demonstrating the trade-off between model size and representational capacity. The Proposed GallNet-X without data augmentation maintained a robust 97.0% accuracy, validating the underlying architecture’s generalizability.

**Table 6:** Comprehensive performance comparison of models

Model	Training Acc. (%)	Testing Acc. (%)	Precision (%)	Recall (%)	F1-Score (%)	Macro Avg	Weighted Avg
DenseNet121	92.78	91.81	92.33	91.44	91.67	0.92	0.92
XceptionNet	90.51	90.44	91.22	90.11	90.56	0.91	0.91
VGG-19	94.04	98.89	97.78	97.78	97.78	0.99	0.99
EfficientNetB0	63.31	75.46	77.11	75.22	75.55	76.0	75.33
MobileNetV2	85.24	93.01	92.89	92.66	92.89	93.00	93.00
Proposed GallNet-X without MSFE	96.90	96.68	97.00	97.00	97.00	0.97	0.97
Proposed GallNet-X without augmentation	96.89	96.86	97.11	96.66	96.88	0.97	0.97
**Proposed MSFE-GallNet-X**	**99.99**	**99.63**	**99.60**	**99.40**	**99.50**	**1.0**	**1.0**

Table 7 summarizes the model complexity and execution time of various CNN architectures, including the proposed GallNet-X variants, where the number of parameters is measured in millions (M), training time in seconds (s), and inference time in milliseconds per step (ms/step). The MSFE-GallNet-X model demonstrates a favorable trade-off between parameter count and computational time. Compared to deeper models such as VGG-19 and lighter models like MobileNetV2, the proposed architecture maintains competitive training and inference performance with substantially fewer parameters, indicating its suitability for deployment in resource-constrained clinical environments. These findings highlight that the MSFE-GallNet-X offers competitive advantages, further establishing its suitability for domain-specific classification tasks in gallbladder analysis.

**Table 7:** Model complexity and execution time comparison

Model	Params (M)	Training Time (s)	Inference Time (ms/step)
DenseNet121	8.61	386	367
XceptionNet	22.96	318	403
VGG-19	74.47	481	536
EfficientNetB0	26.13	239	290
MobileNetV2	26.13	209	259
Proposed GallNet-X without MSFE	0.87	216	223
**Proposed MSFE-GallNet-X**	**1.91**	**349**	**379**

#### Loss

**Fig. 6:**
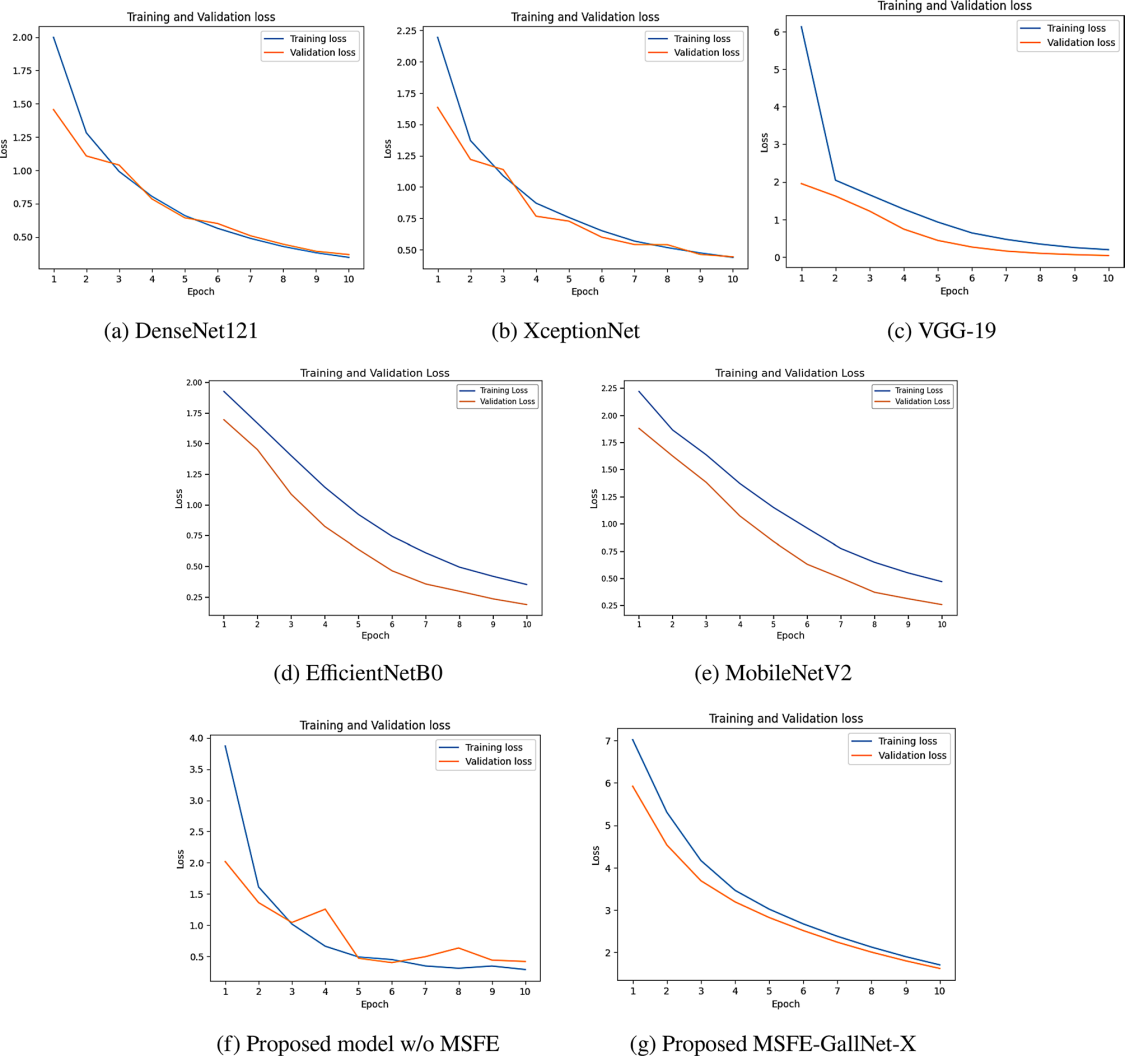
Loss of the models with respect to the number of epochs

To evaluate model performance beyond accuracy, we analyzed the training and validation loss for each of the seven models, such as DenseNet121, XceptionNet, VGG-19, EfficientNetB0, MobileNetV2, GallNet-X without multi-scale feature extraction, and the proposed MSFE-GallNet-X over 10 epochs. All the models exhibited a steady reduction in loss, indicating effective learning throughout the training process. The DenseNet121 model demonstrated a consistent decrease in loss, ending with a training loss of approximately 0.33 and a validation loss of approximately 0.35. XceptionNet showed rapid loss minimization, achieving a training loss of approximately 0.43 and a validation loss slightly under 0.45, indicating robust generalization. VGG-19 followed with impressive performance, reducing training and validation losses to approximately 0.21 and 0.10, respectively, which aligns with its high accuracy. EfficientNetB0 concluded with a training loss of 0.63 and a validation loss of 0.76, whereas MobileNetV2 achieved lower losses of 0.40 (training) and 0.25 (validation), indicating comparatively better generalization. Figure 6 shows the losses of the deep learning models with respect to the number of epochs.

Our proposed MSFE-GallNet-X models displayed competitive and promising results compared to these general-purpose architectures. GallNet-X without MSFE achieved a training loss of approximately 0.31 and a validation loss of 0.47, providing a solid baseline for our custom architecture. However, the most notable improvement emerged with the inclusion of multi-scale feature extraction in the MSFE-GallNet-X. This model achieved the lowest loss values, with a training loss of approximately 1.84 and validation loss of 1.71. The significant reduction in loss with MSFE underscores the advantage of capturing multi-scale features, enabling enhanced feature learning and better alignment between training and validation loss. Figure 6 shows the training and validation loss curves of the five models.

#### Precision, recall and F1-score

Table 6 presents the performance metrics of the various models evaluated based on the precision, recall, and F1-score. Among the models, the proposed MSFE-GallNet-X achieved the highest performance with a precision of 99.60%, recall of 99.40%, and F1-score of 99.50%. GallNet-X without the Multi-Scale Feature Extraction component also performs strongly, with all metrics at 97.0%. VGG-19 ranked closely with a balanced score of 97.78% across all metrics, outperforming DenseNet121 and XceptionNet, which achieved lower yet competitive scores. MobileNetV2 also delivered strong results, attaining a precision of 92.89%, recall of 92.66%, and F1-score of 92.89%, making it the most effective lightweight architecture evaluated. In contrast, EfficientNetB0 lagged significantly, with precision, recall, and F1-score around 75%, indicating limited suitability for the task. The proposed GallNet-X without data augmentation maintained near-parity with its fully trained counterpart, achieving an F1-score of 96.88%, which reflects the underlying model robustness even in the absence of augmentation. These results highlight the effectiveness of the MSFE-GallNet-X, especially with the inclusion of MSFE.

#### Class-wise classification report

Table 8 presents the precision, recall, and F1-score for each gallbladder-related condition classified by the proposed model. The model demonstrates strong performance across all classes, indicating high reliability in distinguishing between various gallbladder abnormalities.

**Table 8:** Classification report for gallbladder conditions

Class	Precision	Recall	F1-score
1. Gallstones	0.98	1.00	0.99
2. Abdomen and retroperitoneum	1.00	0.99	1.00
3. Cholecystitis	1.00	1.00	1.00
4. Membranous and gangrenous cholecystitis	1.00	1.00	1.00
5. Perforation	1.00	0.99	1.00
6. Polyps and cholesterol crystals	0.99	1.00	1.00
7. Adenomyomatosis	1.00	0.99	1.00
8. Carcinoma	1.00	1.00	1.00
9. Various causes of gallbladder wall thickening	1.00	0.99	1.00
**Accuracy**			1.00
**Macro Avg**	1.00	1.00	1.00
**Weighted Avg**	1.00	1.00	1.00

#### Confusion matrix

**Fig. 7:**
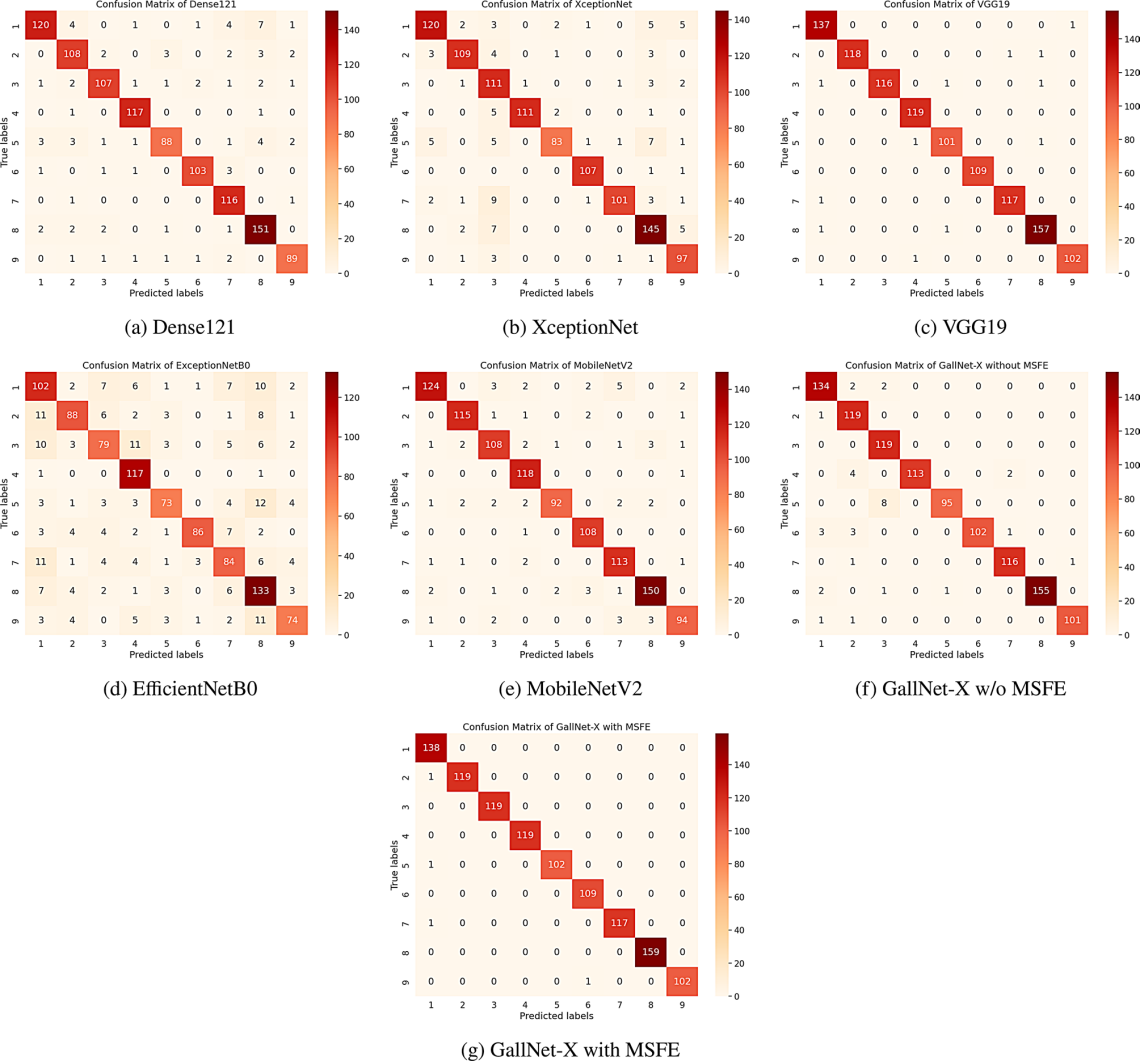
Confusion matrices of the deep learning models used in the study

The visualized generated confusion matrices for multiple deep learning architectures, including DenseNet121, XceptionNet, VGG-19, MobileNetV2, EfficientNetB0, GallNet-X without multi-scale feature extraction, and our proposed MSFE-GallNet-X with MSFE. A comparative analysis of these matrices demonstrated varying performance levels across the different architectures. DenseNet121 and XceptionNet exhibited moderate performance, particularly in cases of membranous cholecystitis and gallbladder wall thickening. VGG-19, while performing better than the previous two models, exhibited some misclassifications between polyps and early-stage carcinomas. MobileNetV2 demonstrated performance closely aligned with DenseNet121, correctly identifying most categories with relatively fewer misclassifications. In contrast, EfficientNetB0 showed the weakest performance among all models, with notable misclassifications across multiple classes, indicating its limited effectiveness for this classification task. The basic GallNet-X without feature extraction demonstrated improved accuracy but still showed confusion between similar pathological conditions. Our proposed MSFE-GallNet-X with multi-scale feature extraction emerged as the superior model, achieving the highest accuracy across all nine categories. The matrix for MSFE-GallNet-X showed particularly strong performance in identifying gallstones, acute cholecystitis, and perforation cases, with minimal misclassifications. The model’s capability to differentiate between adenomyomatosis and carcinoma also showed marked improvement compared with other architectures. Even in challenging cases where gallbladder wall thickening presented with various etiologies, MSFE-GallNet-X maintained robust classification accuracy. These results, visualized through confusion matrices, quantitatively demonstrate the effectiveness of incorporating multi-scale feature extraction in improving diagnostic accuracy across the spectrum of gallbladder diseases. Figure 7 shows the confusion matrices generated by the other models and our proposed MSFE-GallNet-X model.

### State of the art comparison

Table 9 compares various state-of-the-art models applied to gallbladder and related medical datasets. While our primary focus was on gallbladder disease classification, we selected comparative studies that used datasets with characteristics closely aligned with ours, such as grayscale ultrasound or CT images, to ensure a meaningful and contextually relevant evaluation. Zou et al. achieved an accuracy of 92.77% on the WBC Dataset without using XAI [41]. Gupta et al. applied a deep learning technique to the GBCU Dataset, achieving an accuracy of 91.0% with the integration of Explainable AI techniques [42]. The model by Kim et al., using the Gallbladder Polyp Dataset, achieved an accuracy of 87.61% without incorporating XAI [25]. Babuji et al. used the Kaggle Dataset to reach an accuracy of 94.07%, also without XAI [43], whereas Wang et al.’s model achieved 86.50% accuracy on data from the Physical Examination Center of Shengjing Hospital Affiliated to China Medical University, similarly without XAI [44]. These comparisons highlight the varying accuracies across datasets and underscore the potential impact of XAI, as observed in Gupta et al.’s work.

**Table 9:** State of the art

Ref	Model	Dataset	No. of Images	Result (%)	XAI
[41]	ICAFF-MobileNetv2	WBC Dataset	237	Acc: 92.77	No
[42]	GBCNet	GBCU Dataset	1,255	Acc: 91.0%	No
[42]	MedViT	GBCU Dataset	1,255	Acc: 73.0%	No
[25]	Ensemble Model	Gallbladder Polyp Dataset	1,460	Acc: 87.61%	No
[43]	Novel K & Novel Fuzzy C	Kaggle Dataset	5,350	Acc: 94.07%	No
[44]	MLP & J4	Shengjing Hospital Dataset	4,215	Acc: 86.50%	No
[45]	VGG-16 with U-net	Gallbladder Diseases Dataset	57,051	Acc: 95.3%	No
[46]	GBCHV-Trans + attention	Gallbladder Diseases Dataset	1,255	Acc: 96.21%	Yes
[47]	CBIR System	UIdataGB	10,692	AP: 94.00%	No
**Our Model**	**MSFE-GallNet-X**	**UIdataGB**	**10,692**	**Acc: 99.63%**	**Yes**

### Explanibale AI (XAI)

Explainable Deep Learning enhances the transparency of AI models in medical imaging, providing healthcare professionals with clear insights into the decision-making process and enabling trust in AI-powered diagnostic tools. Among the various explainable AI techniques, Gradient-weighted Class Activation Mapping (Grad-CAM) and Local Interpretable Model-Agnostic Explanations (LIME) have emerged as a prominent approach in medical imaging analysis, particularly for identifying gallbladder diseases such as cholecystitis and gallstones. This visualization technique enhances diagnostic accuracy by emphasizing the specific areas that influence the model’s decisions and providing medical professionals with valuable visual evidence for informed clinical decision-making.

In this study, we implemented the Grad-CAM and LIME explainable deep learning techniques. These methods provide visual explanations for our MSFE-GallNet-X model’s classifications by highlighting the specific regions in gallbladder ultrasound images that influenced the model’s diagnostic decisions.

#### GRAD-CAM

Grad-CAM is an explainable deep learning technique that increases model transparency by visualizing key regions that influence predictions, which is particularly useful in medical imaging. In ultrasound examinations for gallbladder disease detection, Grad-CAM highlights areas of interest, helps clinicians validate the focus of AI models, and promotes confidence in AI-assisted diagnoses.

**Fig. 8:**
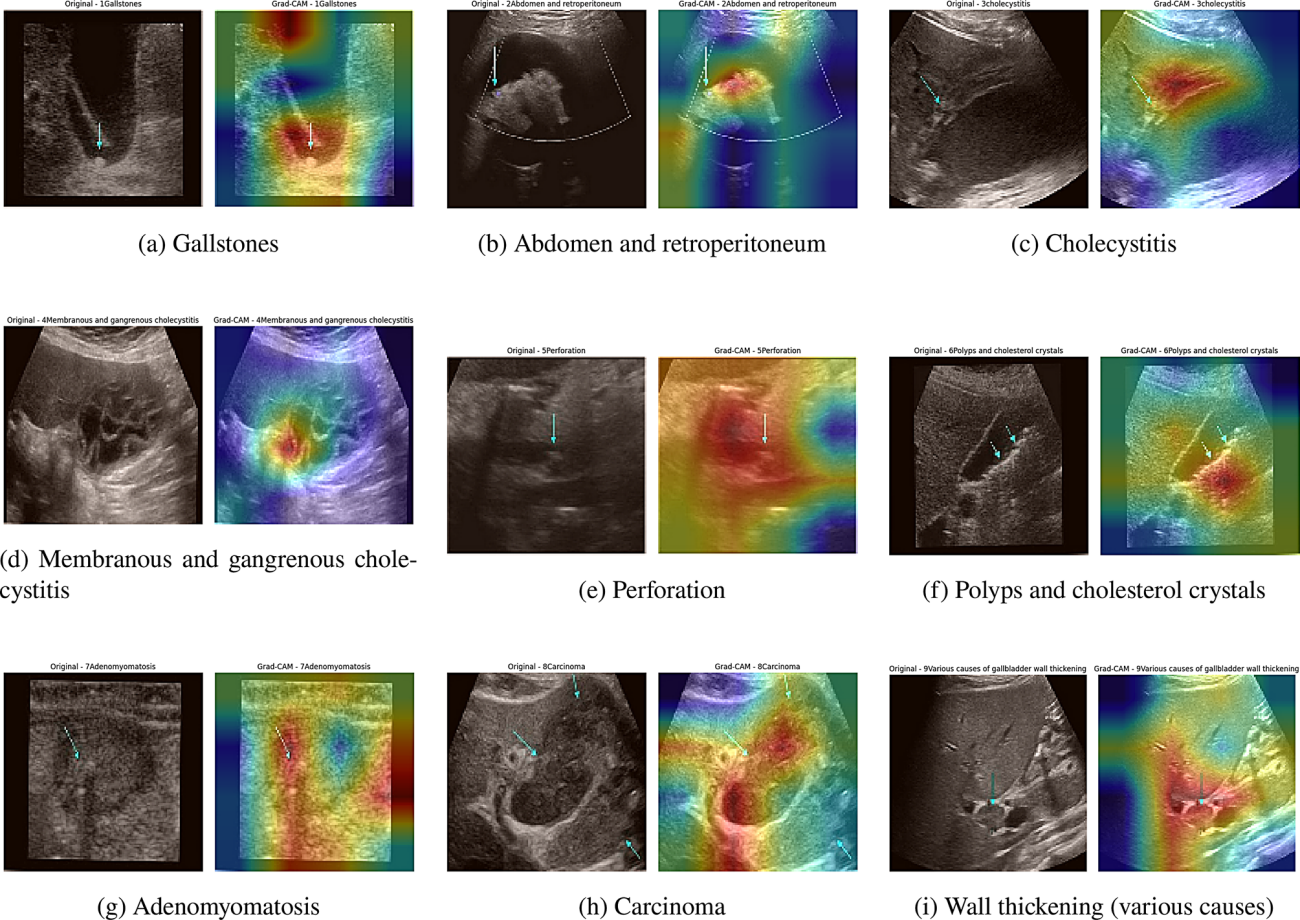
Comparison of different models using GRAD-CAM heatmaps across various gallbladder conditions

In our MSFE-GallNet-X model, we implemented Grad-CAM to provide interpretable results for gallbladder classification tasks. Using Grad-CAM, we created visual explanations that highlight specific regions in the ultrasound images of the gallbladder, confirming that MSFE-GallNet-X’s focus is on medically significant areas. Notably, the model consistently highlighted regions indicated by arrows pointing to areas of involvement, underscoring the reliability of its attention to clinically relevant areas. This capability supports diagnostic accuracy and improves clinical reliability by providing healthcare professionals with insights into how the MSFE-GallNet-X reaches its conclusions. The successful integration of Grad-CAM into MSFE-GallNet-X demonstrates its potential as a valuable tool in AI-driven medical imaging, helping bridge the gap between advanced model performance and real-world clinical usability. Figure 8 shows the GRAD-CAM heatmaps generated by our proposed model on the ultrasound images of the gallbladder.

#### LIME

LIME is another explainable AI technique designed to increase model interpretability by approximating complex model behavior with interpretable, local surrogate models. By perturbing input data and observing resulting predictions, LIME identifies which features contribute most to the model’s decision for a specific instance. This localized insight is particularly beneficial in medical imaging, where understanding individual predictions can help clinicians validate outcomes and maintain trust in diagnostic support tools.

**Fig. 9:**
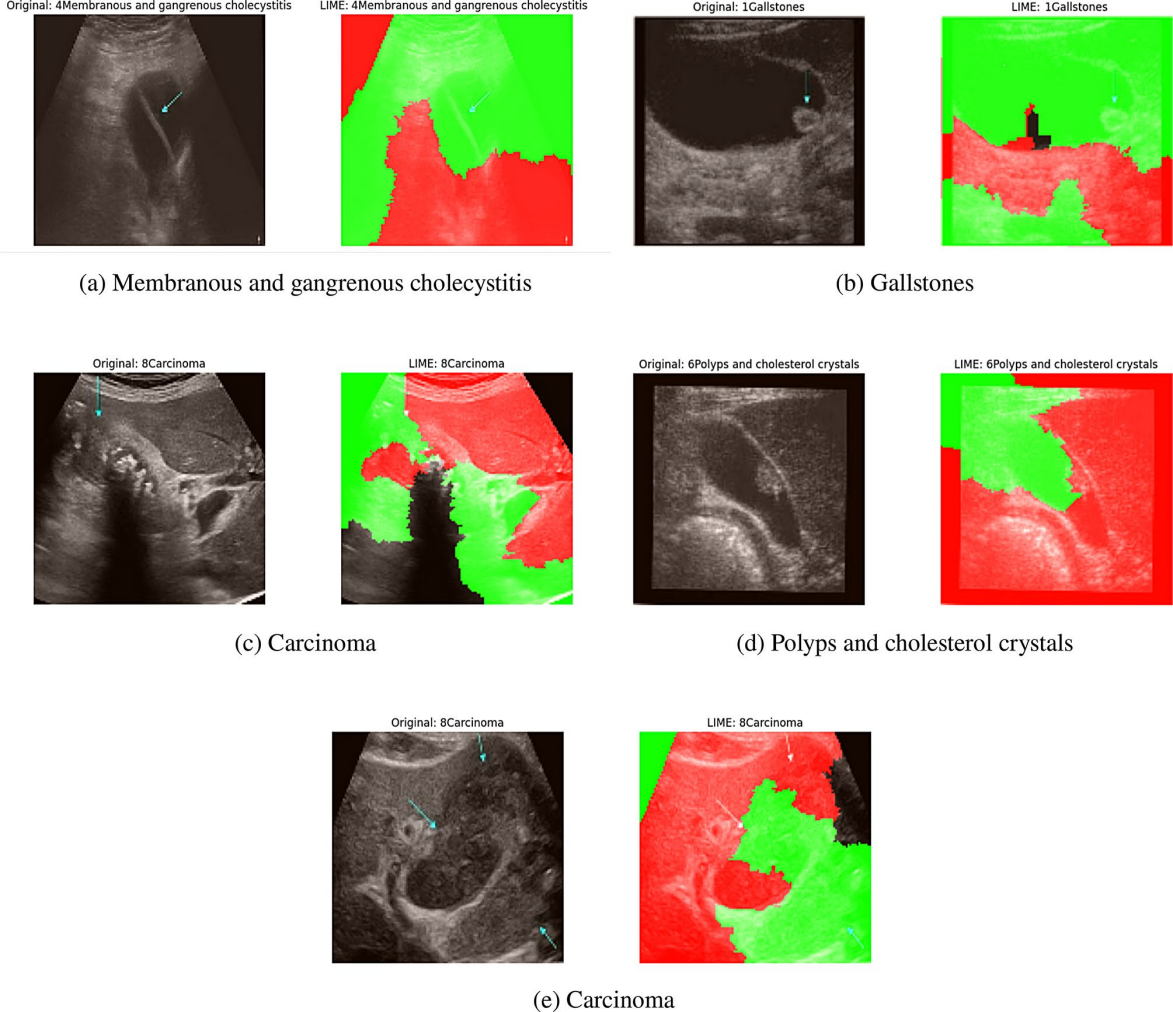
Comparison of different models with LIME heatmaps

In our implementation of LIME for the MSFE-GallNet-X model, the visual explanations are color-coded to reflect the influence of different regions on the model’s decision-making process. Specifically, areas highlighted in green indicate regions that have a significant positive contribution to the model’s prediction, while areas marked in red denote regions that negatively impact the model’s confidence in its prediction. In the context of our medical ultrasound images, the green regions typically align with clinically relevant areas, such as arrow markings or localized defects, which are indicative of pathological findings. This visual alignment helps verify that the model is focusing on medically significant regions when making predictions. Figure 9 illustrates the LIME-based explanations generated for gallbladder ultrasound images using our proposed model.

### Discusions

The lower accuracy of pre-trained models such as DenseNet121 and VGG-19, despite similar experimental conditions, originates from their inherent training bias. These models are optimized for object recognition in natural RGB images (e.g., ImageNet) and often emphasize color gradients, texture continuity, and large-scale structures. Such features are largely irrelevant or even misleading in grayscale ultrasound imaging. In contrast, MSFE-GallNet-X was trained from scratch and benefits from domain-specific augmentation and architecture, allowing it to learn from the fine-grained and noisy patterns characteristic of gallbladder ultrasound.

The MSFE-GallNet-X is a specialized architecture designed specifically for gallbladder disease classification, leveraging a Multi-Scale Feature Extraction mechanism. This design enables MSFE-GallNet-X to effectively capture both fine-grained and broader contextual features within the ultrasound images, improving its resilience to noise and enhancing its ability to focus on medically relevant areas. The MSFE allows MSFE-GallNet-X to filter out irrelevant information more accurately and isolate diagnostic features, an advantage that pre-trained general-purpose models lack. As a result, MSFE-GallNet-X achieves superior accuracy and generalization by aligning closely with the specific demands of gallbladder ultrasound imaging, surpassing the less specialized pre-trained architectures despite their complexity.

Initially, GallNet-X showed irregular spikes without an MSFE block. Subsequently, the integration of the MSFE block with the CNN supported the model to capture features at different resolutions, which made it less reliant on specific patterns or details that may not generalize well to new data. By analyzing the features at various scales, the model became better at learning the essential characteristics of the data rather than noise, which helped mitigate overfitting. Training and validation accuracy/loss curves (Fig. 5(e), 6(e)) show that the model stabilized early, confirming convergence. Although training was set for 10 epochs, early stopping prevented overfitting and saved training time.

While the confusion matrix of the proposed model shows near-perfect classification across all nine categories, this result reflects the dataset’s clean acquisition protocol and balanced class distribution. Despite the model’s excellent accuracy on the test set, we acknowledge the risk of overinterpretation. The dataset was well-structured, balanced, and stratified. While these conditions support strong model performance, we caution that these results may not reflect broader clinical variability. We emphasize this as a limitation to ensure responsible interpretation.

Despite the high accuracy, we observed minor misclassifications between visually similar categories, which can present slight differences in grayscale ultrasound. For example, some misclassifications made by our proposed model (shown in Fig. 1^0^) can be attributed to the presence of stone-like structures in images from the Abdomen and Retroperitoneum, Perforation, and Adenomyomatosis classes. Gallstones typically appear as irregular or round stone-like formations. Interestingly, these three classes also exhibit similar stone-like structures attached to membrane cells, which may visually resemble gallstones. This similarity likely led the model to confuse these classes, resulting in misclassifications. This pattern of confusion is also reflected in the confusion matrix. To address this, future iterations of MSFE-GallNet-X could integrate enhancements such as spatial or channel attention mechanisms (e.g., SE blocks or CBAM), adaptive MSFE scaling, or multi-branch dilated convolutions. These improvements would enable the model to focus more selectively on diagnostically relevant regions and better distinguish overlapping patterns. Additionally, class-specific tuning, such as using focal loss or hard example mining, could further improve sensitivity for challenging categories.

**Fig. 10:**
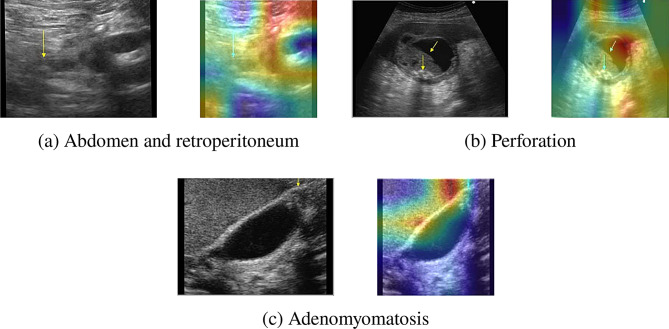
Misclassification of three different classes and their GRAD-CAM output

Also, LIMEs perturbation-based approach may occasionally generate unrealistic artifacts in ultrasound imaging. Similarly, Grad-CAM can sometimes highlight non-pathological regions, including areas unrelated to the actual lesion. However, clinical feedback from expert radiologists confirmed that in the majority of cases, both techniques successfully localized diagnostically relevant regions. Although we reported weighted F1-score, we acknowledge that oversampling or class-weighted losses could improve robustness and plan to explore these in future work. We acknowledge that real-world data may contain noise, variability, or artifacts. In future work, we plan to test our model on diverse multimodal datasets to assess generalizability. In real-world clinical settings, the visual distinction between classes like carcinoma and adenomyomatosis may be less clear, potentially leading to higher misclassification rates.

## Conclusions

This study presented MSFE-GallNet-X, a specialized deep-learning architecture for the accurate classification of gallbladder diseases using multi-scale feature extraction combined with explainable AI techniques. Our model demonstrated exceptional performance, achieving 99.63% accuracy on a large dataset of high-resolution ultrasound images representing nine classes of gallbladder diseases. By utilizing Grad-CAM and LIME for interpretability, MSFE-GallNet provides valuable insight into its decision-making process, assisting healthcare professionals in validating model predictions and improving diagnostic reliability. This study highlights the potential of MSFE-GallNet-X as a powerful and interpretable tool for clinical applications in the diagnosis of gallbladder disease. Although MSFE-GallNet-X shows promising results, there are still some challenges, particularly the lack of publicly available datasets that limit our ability to estimate the model’s performance across diverse data. In addition, challenges occur in interpretability because the model occasionally highlights incorrect regions in ultrasound images, which can lead to misinterpretation. To address this issue, the XAI components need to be refined to further improve the prediction accuracy. In future work, we aim to enhance the generalizability and clinical applicability of MSFE-GallNet-X by incorporating diverse, multi-institutional datasets that capture a broader range of demographic and imaging variations. To ensure robust and leakage-free evaluation, we plan to implement patient-level data splitting and K-Fold cross-validation with patient-wise stratification. We also intend to investigate domain adaptation and anomaly detection techniques to improve performance on low-quality or unseen data. Finally, integrating multimodal data sources such as clinical records and complementary imaging modalities may further boost diagnostic accuracy and extend the model’s utility in real-world healthcare settings.

## Data Availability

This dataset is publicly available here: https://data.mendeley.com/datasets/r6h24d2d3y/2.
